# Anti-Inflammatory Potential of Daturaolone from *Datura innoxia* Mill.: In Silico, In Vitro and In Vivo Studies

**DOI:** 10.3390/ph14121248

**Published:** 2021-11-30

**Authors:** Muhammad Waleed Baig, Humaira Fatima, Nosheen Akhtar, Hidayat Hussain, Mohammad K. Okla, Abdulrahman Al-Hashimi, Wahidah H. Al-Qahtani, Hamada AbdElgawad, Ihsan-ul Haq

**Affiliations:** 1 Department of Pharmacy, Quaid-i-Azam University, Islamabad 45320, Pakistan; mwbg7@yahoo.com (M.W.B.); hfchughtai@qau.edu.pk (H.F.); 2 Department of Biological Sciences, National University of Medical Sciences, Rawalpindi 46000, Pakistan; nosheenakhtar@numspak.edu.pk; 3 Leibniz Institute of Plant Biochemistry, Department of Bioorganic Chemistry, Weinberg 3, D-06120 Halle (Saale), Germany; hidayat.hussain@ipb-halle.de; 4 Botany and Microbiology Department, College of Science, King Saud University, P.O. Box 2455, Riyadh 11451, Saudi Arabia; malokla@ksu.edu.sa (M.K.O.); alhashimi@ksu.edu.sa (A.A.-H.); 5 Department of Food Sciences & Nutrition, College of Food & Agriculture Sciences, King Saud University, Riyadh 11451, Saudi Arabia; wahida@ksu.edu.sa; 6 Integrated Molecular Plant Physiology Research (IMPRES), Department of Biology, University of Antwerp, 2020 Antwerp, Belgium; hamada.abdelgawad@uantwerpen.be

**Keywords:** *Datura innoxia*, daturaolone, terpenoid, anti-inflammatory, NF-*κ*B, nitric oxide

## Abstract

Exploration of leads with therapeutic potential in inflammatory disorders is worth pursuing. In line with this, the isolated natural compound daturaolone from *Datura innoxia* Mill. was evaluated for its anti-inflammatory potential using in silico, in vitro and in vivo models. Daturaolone follows Lipinski’s drug-likeliness rule with a score of 0.33. Absorption, distribution, metabolism, excretion and toxicity prediction show strong plasma protein binding; gastrointestinal absorption (Caco-2 cells permeability = 34.6 nm/s); no blood–brain barrier penetration; CYP1A2, CYP2C19 and CYP3A4 metabolism; a major metabolic reaction, being aliphatic hydroxylation; no hERG inhibition; and non-carcinogenicity. Predicted molecular targets were mainly inflammatory mediators. Molecular docking depicted H-bonding interaction with nuclear factor kappa beta subunit (NF-κB), cyclooxygenase-2, 5-lipoxygenase, phospholipase A2, serotonin transporter, dopamine receptor D1 and 5-hydroxy tryptamine. Its cytotoxicity (IC_50_) value in normal lymphocytes was >20 µg/mL as compared to cancer cells (Huh7.5; 17.32 ± 1.43 µg/mL). Daturaolone significantly inhibited NF-κB and nitric oxide production with IC_50_ values of 1.2 ± 0.8 and 4.51 ± 0.92 µg/mL, respectively. It significantly reduced inflammatory paw edema (81.73 ± 3.16%), heat-induced pain (89.47 ± 9.01% antinociception) and stress-induced depression (68 ± 9.22 s immobility time in tail suspension test). This work suggests a possible anti-inflammatory role of daturaolone; however, detailed mechanistic studies are still necessary to corroborate and extrapolate the findings.

## 1. Introduction

Inflammation is a major response to tissue injury which is fabricated to maintain homeostasis by returning the tissue to its pre-injury state. However, deregulated inflammatory processes cause many human diseases such as brain, cardiovascular and bowel diseases, diabetes, arthritis and cancer. Despite the nature of pathological conditions, all inflammatory processes are governed by a common mechanism that essentially includes the activation of inflammatory pathways, followed by the release of inflammatory markers [1]. Among many such markers, tumor necrosis factor (TNF-α), play a significant role in the inflammation cascade of cytokine network by affecting vasodilatation, edema formation, blood coagulation via leukocyte adhesion, oxidative stress generation and indirect induction of fever. Another important inflammatory mediator in the cascade is nitric oxide (NO), the overproduction of which causes vasoconstriction, inflammation, tissue damage and neurodegenerative disorders [2]. Therefore, the exploration of leads with therapeutic value against inflammatory mediators would be worth pursuing for the management of pathologies which are consequential of direct or indirect deleterious actions of dysfunctional inflammation.

**Scheme 1:**
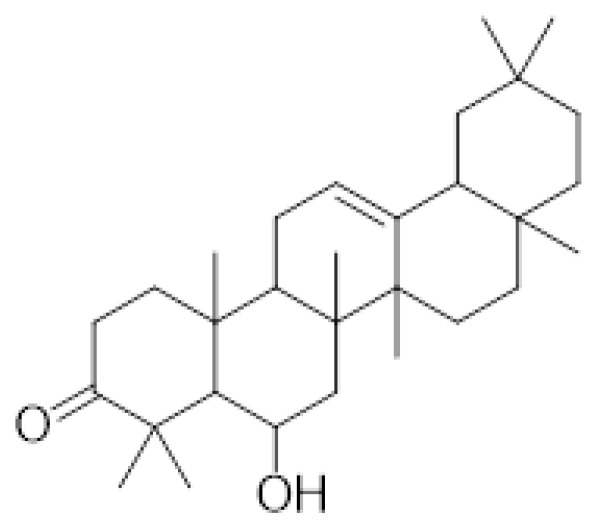
Structure of Daturaolone.

Natural products have historical contributions in drug discovery. Plant-sourced pentacyclic oleanane triterpenoids are a class of compounds that, unlike conventional cytotoxic agents, are multifunctional that specifically aim to act on unique molecular targets in signal transduction pathways of inflammation. Their significant effects on changing the redox state of tissues and cells leads to inflammation [3]. Daturaolone is a pentacyclic oleanane triterpenoid that has been previously reported from *Datura innoxia* Mill [4]. Scientific reports on anti-inflammatory prospects of daturaolone are scarce and earlier study on daturaolone isolated from *P. integerrima* has reported its in vivo anti-inflammatory and antinociceptive potential, which was further corroborated in silico showing its interactions with the COXs receptors only [5]. Bawazeer et al. isolated daturaolone from *D. metel*, wherein, it was tested in various acute in vivo experiments and its role was found to be in relaxation of muscles, gastrointestinal motility and reduction of fever [6]. Furthermore, daturaolone has shown significant α-glucosidase and β-secretase inhibitory activity and has exhibited a potent antibacterial and antifungal spectrum [7,8]. This study is portion of our continuing research on isolation and biological activity determination from *D. innoxia*. This present paper highlights the biological potential of its bioactive metabolite, daturaolone (Scheme 1), against inflammation and related disease outcomes, i.e., cancer, pain and depression. To the best of our knowledge, its detailed anti-inflammatory prospects in silico, in vitro and in vivo have not been described hitherto.

## 2. Results and Discussion

### 2.1. Daturaolone Was Predicted as a Drug-like Compound

Daturaolone complies with Lipinski’s rule. It passed three out of four properties of the mentioned rule with a bioavailability score of 0.55 (Table 1). Its drug-likeliness score (0.33; Supplementary Figure S2) was found to be better than some trial and marketed drugs used as a comparative reference such as withaferin A (0.36), psychoactive tetrahydrocannabinol (−0.01), anti-tumor cisplatin (−1.12) and antiepileptic valproic acid (−0.02), according to the database. This brings daturaolone in line with approved drugs based on its structural properties and suggests it a suitable candidate to proceed investigation into its pharmacological properties.

### 2.2. Absorption, Distribution, Metabolism, Excretion and Toxicity (ADMET) of Daturaolone

Pharmacokinetic specifications address a compound’s accessibility to the active site where it will be befitted for the required pharmacological outcome. PreADMET predicted that daturaolone has high plasma protein binding (PPB), human intestinal absorption (HIA) and moderate Caco-2 cell permeability (Table 1). These parameters propose the compound’s potential as an oral drug. Our in vivo positive results contradict its predicted high PPB. Moreover, molecules which pass BBB via active or passive transport cause toxicity and need to be excluded from brain to maintain its proper functioning [9]. Daturaolone was proposed as neither BBB permeant nor interacting with P-gp, thereby potentially preventing CNS side effects. PreADMET predicted daturaolone to be an inhibitor of CYP2C9 and CYP3A4 cytochrome P450 enzymes whereas, no inhibition was observed through SwissADME. Daturaolone, being an inhibitor of cytochromes enzymes i.e., CYP2C9 and CYP3A4 might be held accountable for increased bioavailability of the tested specimen and can induce both the toxicity risks and beneficial properties of compounds [10]. PreADMET anticipated non-mutagenicity of daturaolone before and after the metabolism of daturaolone by S9 liver homogenous enzymes. Reliable in silico filters for human ether go go related gene (hERG) inhibition remove chemical entities which may be cardiotoxic in the future. [11]. Daturaolone showed low risk of hERG inhibition, which predicts a lesser chance of sudden cardiac arrhythmias. Detailed studies are still required before proving the claims via in silico analysis.

**Figure 1:**
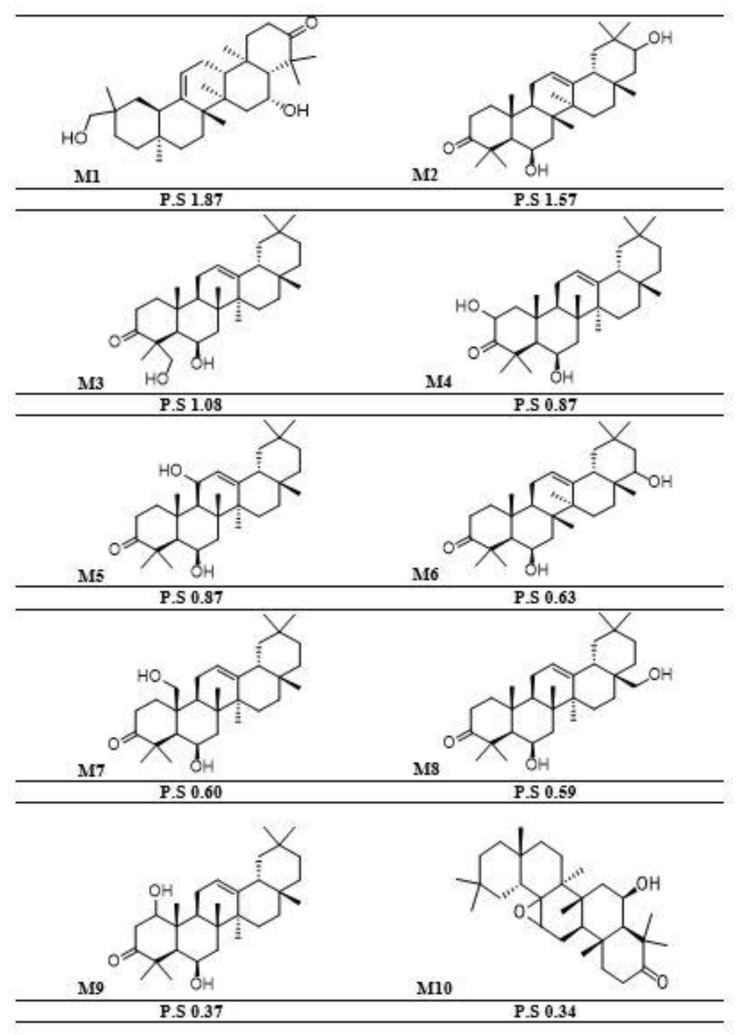
Predicted metabolites of daturaolone. Online web tool GLORY was used to predict Cytochrome P450 metabolite structures of daturaolone Metabolites structures with probability score (P.S) greater than 0.33 i.e., M1 to M10 were drawn in chemDraw and shown in the figure. P.S assigned by GLORY is based on reaction type to form the respective metabolite, atoms involvement probability in the reaction to form that metabolite and if the nature of reaction is common or not. Aliphatic hydroxylation was the metabolic reaction predicted to form all shown metabolites.

Chemical reactions involved in metabolites prediction (Figure 1) were primarily Phase-I, which make daturaolone more water-soluble, thus ensuring renal clearance. The major predicted reaction was aliphatic hydroxylation, while others included alkyl dehydrogenation, alcohol oxidation, deformylation and aliphatic hydroxylation with allylic rearrangement 1 reactions. The literature shows that in silico metabolism estimation predicts active/inactive metabolites formed via hepatic/extra-hepatic models [12]. However, real-time comprehensive metabolism estimation is proposed for future analysis.

### 2.3. Daturaolone Can Interact with Multiple Molecular Targets

Daturaolone was found to exhibit therapeutic potential against various interlinked disorders either via interaction with inflammatory mediators or with disease-specific molecular targets (Table 2). Pathogenesis of inflammation and its related outcomes i.e., carcinogenesis, pain [13] and depression [14] involves nitric oxide synthesis and metabolism of arachidonic acid. In inflammation, release of arachidonic acid from membrane phospholipids is facilitated by phospholipase A2 (PLA2). Cytochrome P450, lipoxygenases (LOX) and cyclooxygenases (COX) cause metabolism of arachidonic acid thus giving rise to three unique pathways in the inflammation cascade. Modulating the metabolism of arachidonic acid by targeting to inhibit COX and LOX is an effective way to handle inflammation and cancer chemoprevention [15]. Cancer development is promoted by prostaglandins (PGE2) that are mediated especially by COX-2 resulting in increased cellular division and proliferation, boosting angiogenesis, and enabling cells to evade normal cell cycle checkpoints of apoptosis. Moreover, increased PGE2 mediated COX-2 production also cause inflammation induced nociception. Not only the sensitivity of peripheral nerve endings is increased but also the pain centre of central nervous system is also agitated. [13]. Inflammatory cytokines also stimulate NO production in macrophages and many other cells, resulting in dysfunctional abnormalities [15]. Besides treating inflammation, NO production impeding entities are also a treatment for depression. Increased NO production is due to glutamate released during altered neuronal signaling in depression via NMDA receptor activation [14]. In the current study, daturaolone was predicted to interact with the above-mentioned inflammatory targets, thereby providing its possible research avenue for inflammatory disorders. 

Predicted antinociceptive targets are known to alleviate inflammatory pain and include cannabinoid receptor 1, predominantly, and non-cannabinoid receptors, e.g., PPAR alpha and 5-HT 1B [16]. Daturaolone was also found to interact with other mediators of depression besides NO. Monoamine theory of depression states that the depression is a result of stress induced dysfunction in neurotransmission, specifically the monoamines that are involved in transmission of signals i.e., norepinephrine, dopamine and serotonin [14]. Multiple molecular targeting by daturaolone is also in conformation with earlier studies where terpenoids display interrelated antiangiogenic, anti-inflammatory, antinociceptive and antidepressant effects. The efficacy of a compound differs with respect to the various activities. A single compound might have a potential to treat different disease outcomes from different angles in modern disease-alleviating strategies [17]. All databases compare query compound to a library of molecules which have proven interaction with molecular targets of different organisms.

### 2.4. Predicted Cytotoxicity in Cancer Cell Lines 

Tumor cells, particularly leukemic, pancreatic, hepatic, gastric and thyroid, were predicted to be moderately susceptible to daturaolone assessed through CLC-Pred (Table 2). These results reinforce our previous in silico analysis illustrating the interaction with inflammatory mediators. The discovery and formulation of safer novel drugs is vital to tackle morphologically, histologically and genetically divergent cancer cell lines. CLC-Pred compares query compound with the real time cell line cytotoxicity data of the same kind of chemical entities exposed to 278 cancer cell-lines [18].

### 2.5. Molecular Docking Analysis

**Figure 2:**
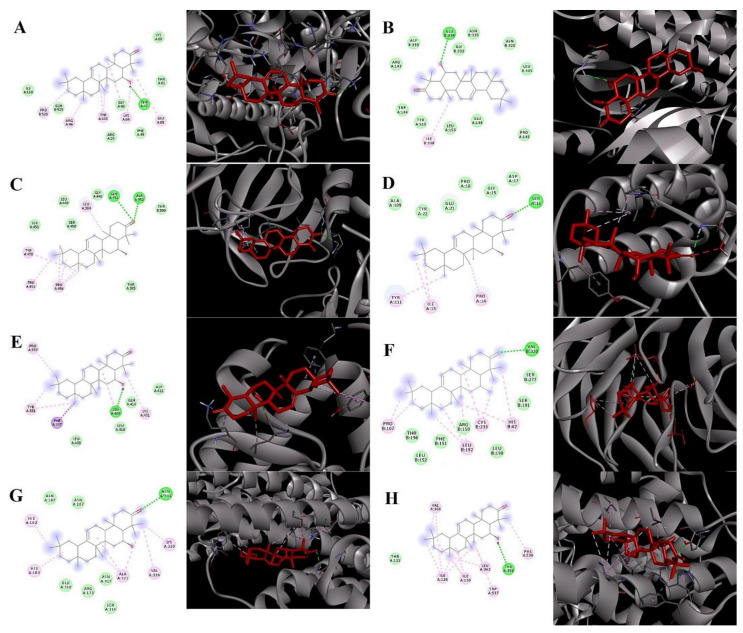
Two dimensional (2D) and three dimensional (3D) visual depiction of daturaolone linking or bonding with protein pockets of different proteins, respectively. COX-2 (**A**), 5-LOX (**B**), NF-κB (**C**), PLA_2_ (**D**), Estrogen receptor (**E**), Dopamine D1 (**F**), 5HTP (**G**) and serotonin transporter (**H**).

The molecular docking approach shows ligand and protein-binding site interactions at the atomic level. Biological potentials of daturaolone in inflammatory disorders were strengthened by this approach. Daturaolone formed H-bonding interactions with several mediators of the inflammatory pathway, hyperalgesia and depression, i.e., COX-2, 5-LOX, NF-κB, dopamine receptor 1A, phospholipase A2, estrogen receptor, 5-hydroxytryptamine and serotonin transporter (Figure 2), the binding energies being −9.2, −9.5, −8.3, −8.6, −7.9, −7.3, −7.9 and −8.8 kcal/mol, respectively. Docking proteins that showed hydrogen bonding interaction with daturaolone have important association with inflammation, depression, hyperalgesia and cancer. H-bonding also ensures stable interaction in the ligand protein complex [19].

COX-2 is involved in cancer development and increased pain intensity [13]. Increased NO production links to depression [14]. LOX causes an increase in IL6 [20]; thus, it is associated with multiple disease outcomes. Similarly, NF-κB linkage to inflammation and cancer is critical [21] and its activation in CNS also links to depression [22]. Antinociceptive targets known to alleviate inflammatory pain involve serotonin receptors [16]. Moreover, impairments in neurotransmission of monoamines, i.e., dopamine and serotonin, cause depression [14].

### 2.6. In Vitro Cytotoxicity of Daturaolone

Considering the cancer cell line cytotoxicity prediction results of in silico experiments, in vitro cytotoxicity or cell viability of daturaolone was assessed in cancer cell lines for hepatocellular carcinoma (Huh7.0 and Huh7.5), prostate cancer (DU145 and PC3), breast cancer (MCF7) and normal isolated lymphocytes (Table 3). Daturaolone (20 µg/mL) induced significant cytotoxicity in hepatic cancer cells, i.e., Huh 7.5 with IC_50_ values of 17.32 ± 1.43 µg/mL. Likewise, a substantial cytotoxicity against DU-145 cell line (IC_50_ = 18.64 ± 2.15 µg/mL) demonstrated its anticancer potential against prostate cancer. Positive controls, cabazitaxel and vincristine, demonstrated IC_50_ values of 4.75 ± 1.12 and 5.62 ± 0.72 µg/mL, respectively (Table 3). A significant biocompatibility and selective toxicity of daturaolone was evident from its low cytotoxicity (29.13 ± 2.57% inhibition) in lymphocytes even at the highest tested concentration, and the results are comparable to vincristine (67.73 ± 1.70%). Analysis of the basic pharmacokinetic mechanisms through which bioactive phytochemicals induce their antineoplastic effects reveal that a multimode panel of molecular targets are involved, including protein kinases, apoptotic and antiapoptotic proteins, transcription factors i.e., Nrf2, Ap1, NF-κB and p53), growth factors i.e., PDGF, TNF, EGF and FGFEGF, TNF, FGF and PDGF), cell division proteins and cell adhesion molecules. Interference with different steps in cell signaling pathways has also been associated as a possible anti-tumor mechanism of phytochemicals. Impairment of cellular apoptosis has been frequently allied with hyperproliferative conditions such as cancer and autoimmune diseases; therefore, cytotoxicity and in vitro whole cell viability assays that quantify cell-death-related phenomena are extensively used for anti-tumor drug development [23].

### 2.7. PK, TNF-α Activated NF-κB and NO Production Inhibition Properties of Daturaolone

Molecular target prediction directed PK, TNF-α activated NF-κB and NO production inhibition assays were performed. Inhibition of aerial hyphae formation by daturaolone was observed. The bald zone of 12.37 ± 2.41 mm (Table 4) surrounding the daturaolone wetted discs depicted comparable results to surfactin control (28.23 ± 2.63 mm) (Table 4). *Streptomyces* require protein kinases for hyphae formation and successful inhibition of PKs results in bald zone phenotypes. One of the most significant governing step of biological system is phosphorylation of tyrosine and/or threonine/serine residues of proteins by various protein kinases. In tumorigenesis, genetic alterations deregulate protein kinases which is the common underlying feature in inflammation and cancer. [24]. The PK inhibition potential of daturaolone suggests it as a suitable candidate for the treatment of inflammation. Streptomyces rely on protein kinases to form aerial hyphae. Samples which halt aerial hyphae formation are considered to be inhibitors of protein kinases. In the present study, this characteristic of *Streptomyces* has been taken advantage of for probable inhibitory potential of test compound [25]. 

Development of inflammation and cancer are reported to be controlled by suppressing the major modulators i.e., nuclear factor-κB (NF-κB) and nitric oxide (NO), respectively. Natural compounds including terpenoids shows that their potential to target cancer and inflammation can provide possible avenue for new discovery [26]. It was observed that daturaolone exhibited significant percent inhibition of TNF-α activated NF-κB with a value of 92.17 ± 5.1% (IC_50_: 1.2 ± 0.8 µg/mL), and the results are in justification with our studies, in which NF-κB was shown to form an interaction with the compound in molecular docking analysis. Detailed mechanistic and molecular studies have revealed the involvement of NF-κB in coordinating the gene expression associated with the cell survival, growth adherence, differentiation, proliferation and inflammation [27]. TNF-α belongs to the proinflammatory cytokines, and this in combination with the release of chemokines leads to the recruitment of different populations of leukocytes seen in inflammatory processes. Furthermore, TNF-α cause over-expression of cyclo-oxygenase 2 (COX-2), subsequently causing an increased production of vasodilatory prostaglandins ultimately leading to vasodilatation, causing redness of skin and heat production. Commercially available TNF- α blocking agents i.e., etanercept, adalimumab and infliximab are used to address severe inflammatory issues like Crohn’s disease, ankylosing spondylitis and rheumatoid arthritis. [28]. 

Many inflammatory mediators are released by macrophages during the chronic course of inflammation, including interferons, cytokines, chemokines, colony-stimulating factors, proteases, eicosanoids, lysozymes, growth factors and nitric oxide (NO). Amongst these mediators, NO is produced from L-arginine endogenously by the proinflammatory enzyme, inducible nitric oxide synthase (iNO_S_), and subsequently results in various diseases including psoriasis, asthma, arthritis, colitis, multiple sclerosis, neurodegenerative disorders and tumor development [29]. Thus, the discovery of novel iNO_S_ or NO production inhibitors may open a novel avenue in anti-inflammatory therapy. In the current study, daturaolone significantly inhibited NO with % inhibition of 84 ± 2.87 and an IC_50_ value of 4.51 ± 0.92 µg/mL. It is comparable to positive control curcumin with IC_50_ value of 2.94 ± 0.74 µg/mL. These results showed that daturaolone, being a potent inhibitor of PK, TNF-α activated NF-κB and NO production, is a noteworthy compound for the management of inflammatory disorders.

**Figure 3:**
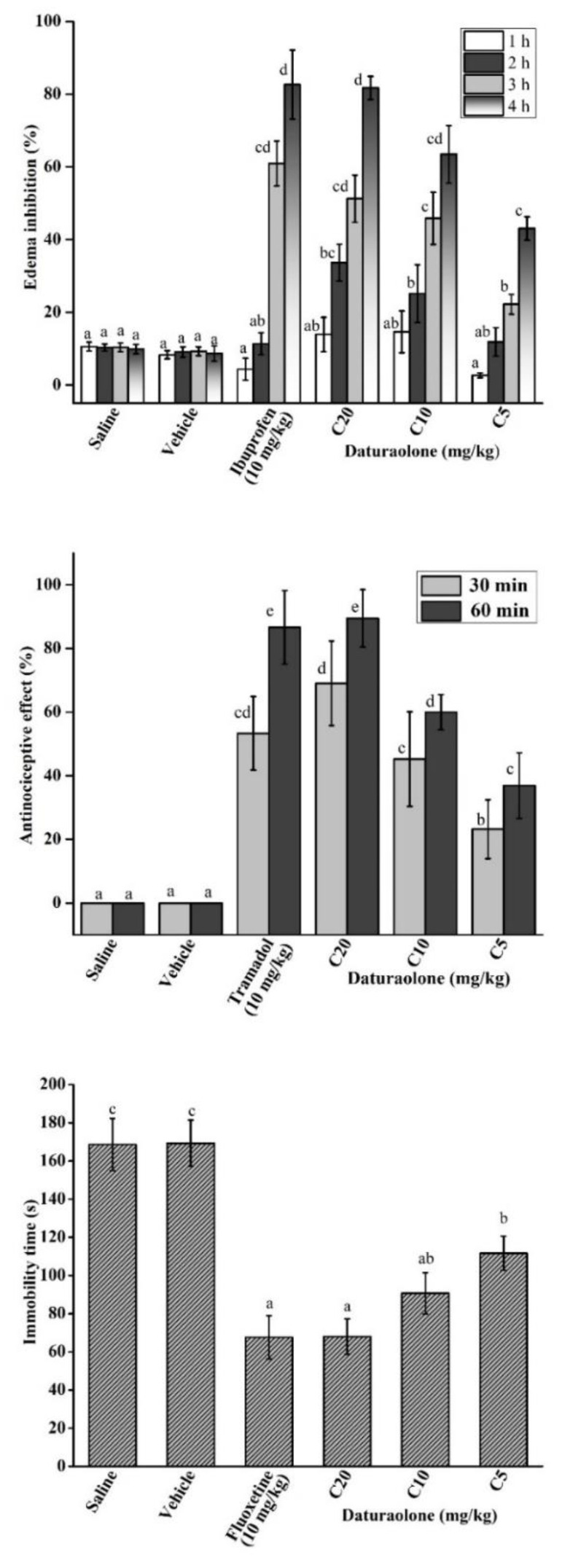
Anti-inflammatory, antinociceptive and antidepressant potential of daturaolone. Anti-inflammatory, antinociceptive and antidepressant effects were assessed by carrageenan-induced paw edema, hot plate and tail suspension methods, respectively. C20, C10 and C5 represents 20, 10 and 5 mg/kg daturaolone. Positive controls used were fluoxetine, tramadol and ibuprofen. Experiment was performed on 6 mice (*n* = 6) and values were calculated as mean ± SD. Means with different letters (^a–d^) are significantly different from one another (*p *< 0.05).

Based on predicted in silico and positive in vitro results, we assessed in vivo anti-inflammatory, antinociceptive and antidepressant activities as these are interlinked in one way or another (Figure 3).

### 2.8. Daturaolone Reduced Inflammation In Vivo

**Figure 4:**
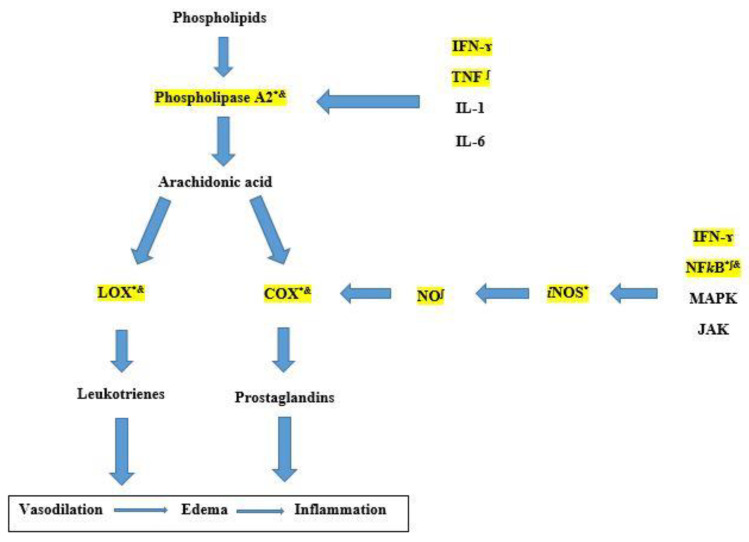
Proposed anti-inflammatory mechanism of action of daturaolone. Targets of daturaolone on inflammation cascade are highlighted, as observed by * molecular target prediction, ^∫^ in vitro assays and ^&^ molecular docking analysis. In the study, daturaolone interacts with TNF-α, phospholipase-A2, COX, LOX and NF-κB, thus disrupting inflammation cascade down the line. It might also inhibit NO generation by directly interacting with *i*NOS or indirectly by interaction with other inflammatory proteins.

Daturaolone (C20) at highest dose of 20 mg/kg gave highest inhibition of edema in moue paw with 81.73 ± 3.16%. Result was somehow equivalent to positive control (ibuprofen) i.e., 82.64 ± 9.49% (Figure 2). In inhibition of inflammation via a carrageenan-induced paw edema model, it has been observed that daturaolone acts on the molecular descriptors involved in second phase of inflammation where prostaglandins such as TNF-α and interleukins such as IL-1, IL-10 and IL-6 are held responsible. [30]. Molecular target prediction, in vitro results and molecular docking analysis also support these findings, where daturaolone was shown to interact with different mediators of inflammatory pathways. A proposed mechanism is shown in Figure 4.

### 2.9. Daturaolone Diminished Thermal Pain 

In clinical settings, pain and depression are the common physical and psychological symptoms, respectively. As a result of inflammation, pain and depression also develops side by side which highlights the involvement of same mediators and following somewhat identical and overlapping pathogenesis [31]. It was observed that tramadol, which was used as positive control, exhibited maximum relief from thermal pain after 1 h with 86.6 ± 11.5% (*p* < 0.05) antinociception (Figure 2). Dose reliant antinociception was observed by daturaolone where maximum activity was depicted by highest dose (C_20_) with 89.47 ± 9.01% antinociception (*p* < 0.05 compared to vehicle control) after 1 h. Some pyrogenic cytokines and prostaglandins activate fever in indirect manner, TNF- α being one of them. It causes stimulation of microglia and endothelial cells to augment prostaglandin E2 production. As a result, thermoregulatory sensitivity via nociceptive receptors in brain is increased. [32]. Similarly, an increase in plasma NO level and its metabolites correlates with pain intensity [33]. Therefore, inhibitors of NO and NF-κB signaling cascade have beneficial antinociceptive effects, with daturaolone being one of them. 

### 2.10. Daturaolone Improved Depression 

In a tail suspension antidepressant assay, daturaolone lessened the movement capability of mice from 169.33 ± 12.04 s in vehicle control group to a maximum of 68 ± 9.22 s (*p* < 0.05) by daturaolone (C_20_) (Figure 2). This lessened movement capability time indicates an improvement in mice depressive behavior after treatment with daturaolone. Comparable results with fluoxetine (positive control) were observed, where immobility time was reduced to 67.66 ± 11.37 s. It has been scientifically proven that the synthesis of cytokines which are responsible for inflammation can incite such neuroendocrine changes which are interpreted as stressors by the brain. It would ultimately induce the hyperactivity of the hypothalamic–pituitary–adrenal axis, which in turn starts producing hormonal products, leading to depression. Moreover, depression and inflammation share some common markers including raised levels of the inflammatory cytokines i.e., C- reactive proteins, TNF- α and interleukins (IL6) [34]. Therefore, it can be postulated that if the isolated compound has potent anti-inflammatory action, then it might be able to treat the depression as well. Serotonin, dopamine and norepinephrine are the critical neurotransmitters associated with neurobiology of major depressive disorder which are mediated by nitric oxide (NO). Targeting nitric oxide (NO) to block or inhibit its production production play a critical role to improve swimming behavior and reduce immobility time in rodents forced swim test, thus displaying antidepressant-like activity [35]. Daturaolone might owe its antidepressant effect to the inhibition of TNF-α activated NF-κB and NO production.

## 3. Materials and Methods 

The methodology used for in silico, in vitro and in vivo analysis is well established and appropriately cited.

### 3.1. Chemicals and Cell Lines

Ibuprofen, cabazitaxel, vincristine, tramadol, ibuprofen, fluoxetine, aspirin and carboxymethyl cellulose (CMC) were purchased from Sigma Aldrich (Taufkirchen, Germany). Solvents i.e., dimethylsulfoxide (DMSO), n-hexane, ethyl acetate and ethanol were purchased from Sigma Aldrich. Unless stated otherwise, almost all solvents and chemicals were purchased from Sigma Aldrich. ISP4 medium was prepared in the laboratory. Distilled and/or deionized water was freshly prepared.

Prostate Cancer ((DU145 (HTB-81) and PC3 (CRL-1435)), breast cancer ((MCF-7 (HTB-22)) and hepatic cancer ((HuH-7 (CCL-185) and HuH-7.5 (PTA-8561) were used in the study. Cancer cells were grown and maintained in Dulbecco’s Modified Eagle Medium (DMEM), Streptomycin sulfate 100 μg/mL, amphotericin Bin Dulbecco’s Modified Eagle Medium (DMEM; 100 μg/mL streptomycin sulfate, 100 IU/mL penicillin G sodium, 0.25 μg/mL amphotericin B 0.25 μg/mL. penicillin G Sodium 100 IU/mL). RPMI-1640 medium (2.2 g/L sodium bicarbonate, pH 7.4) supplemented with 10% *v*/*v* HIFBS (heat inactivated fetal bovine serum) in a humidified incubator (37 °C, 5% CO_2_). Lymphocytes and macrophages were freshly isolated.

### 3.2. Isolation of Daturaolone

*D. innoxia* fruits were collected from Islamabad, Pakistan during the month of June. The collected plant material was authenticated by Prof. Dr. Rizwana Aleem Qureshi, Department of Plant Sciences, Faculty of Biological Sciences, Quaid-i-Azam University Islamabad, Pakistan. A voucher specimen (PHM-525) of the dried plant was archived to herbarium of medicinal plants, Quaid-i-Azam University Islamabad. The compound was isolated via column chromatography and characterized through _1_H, _13_C, HMBC, HSQC, COSY NMRs and GC-MS, which is detailed in the Supplementary Materials (Supplementary Figure S1a–i).

### 3.3. Animals

In the undertaken study, BALB/c mice of 7–8 weeks age and 25–30 g weights from both the sexes. All the necessary guidelines for animal care were followed in conformity with the National Institute of Health, USA guidelines for the care and use of laboratory animals. The mice were housed in the Primate Facility of Faculty of Biological Sciences, Quaid-i-Azam University Islamabad, Pakistan with suitable environmental conditions (23–25 °C, 50% ± 10% relative humidity), standard light/dark conditions. Food and water were provided ad libitum. Utmost care was taken not to harm the animals. An acclimatization time of one week was given before starting the designed experiment. The study was conducted after ethical approval from the Institutional Animal Ethics Committee (letter number # BEC-FBS-QAU2019-135). To avoid mice distress, stringent precautions were taken, and a sufficient number of wooden shavings are evenly spread on the entire floor as bedding.

### 3.4. In Silico Screening

#### 3.4.1. Drug Likeliness Prediction

Drug-likeliness property prediction of daturaolone and drugs was performed using online tools i.e., Molsoft, PreADMET and SwissADME. To calculate drug-likeliness, these online tools follow Lipinski’s, CMC like, lead like, Ghose, Eguan, Muegge, Veber, World Drug Index and MDDR like rules. Drug-likeness score was calculated by Molsoft [36].

#### 3.4.2. Absorption, Distribution, Metabolism, Excretion and Toxicity (ADMET) Profile Prediction

Plasma protein binding, gastrointesitinal absorption (%) i and Caco-2 cells permeability (nm/sec) was calculated by PreADMET. Swiss ADME was used to calculate the values of bioavailability, blood brain barrier permeability, P-gp substrate and water solubility. Swiss ADME utilizes BOILED-EGG method for blood brain barrier permeation prediction. For toxicity prediction, PreADMET calculated the prediction of daturaolone in Ames Test, hERG inhibition analysis and carcinogenicity in mouse and rat. PreADMET checks query compound for toxicity by comparing it with National Toxicology Program and FDA (USA) in vivo two-year carcinogenicity data on rodents [36]. Cytochrome P450 mediated hepatic/extra hepatic metabolites of daturaolone were checked by GLORY web tool [37].

#### 3.4.3. Molecular Target and Cancer Cell Line Cytotoxicity Prediction

Prediction of biological property and molecular targets of daturaolone was performed using Swiss Target Prediction [36], PASS and GUSAR-antitargets software. The aforementioned softwares provide probable molecular targets after comparing two dimensional (2D) and three dimensional (3D) structural similarities of query compound with the available bioactive molecules of the database. Cancer cell lines cytotoxicity prediction was done using PASS CLC-Pred online tool [18].

#### 3.4.4. Molecular Docking Analysis

Molecular target predictions and protein ligand interactions of daturaolone with COX-2, 5-LOX, estrogen receptor, dopamine receptor D1, phospholipase A2, 5-hydroxytryptamine, NF-κB and serotonin 2A receptor and serotonin transporter were determined according to previously stated protocol with slight modifications [38]. Pdb format of protein structures was downloaded from Protein Data Bank (PDB) with corresponding id’s of 3LN1, 3V99, 5FQS, 7JOZ, 3ELO, 6A93, 4G3E and 5I6X. 

### 3.5. In Vitro Studies

#### 3.5.1. Cytotoxic Activity

MTT (3-(4,5-dimethylthiazol-2-yl)-2,5-diphenyl tetrazolium bromide) assay was employed to determine the in vitro cytotoxicity of daturaolone (20 µg/mL; C20) against various cancer cell lines (72 h) i.e., hepatic (HuH-7.0 and HuH-7.5), prostate (DU-145 and PC3), breast cancer (MCF-7) and freshly isolated human lymphocytes (24 h) [36]. Positive controls (20 µg/mL) used were cabazitaxel and vincristine. Negative control employed was 1% DMSO. Cell lines in which there was ≥ 50% cytotoxicity were further tested at two- fold serial dilutions i.e., 2.5, 5 and 10 µg/mL. Lymphocyte isolation via lymphoprep required sampling of blood form a healthy volunteer. This study was also approved by the Institutional Review board of Quaid-i-Azam University (letter number # BEC-FBS-QAU2019-135). Written informed consent was obtained from the participant.

#### 3.5.2. Protein Kinase (PK) Inhibition Assay

PK inhibition potentiality of daturaolone (20 μg; C20) was determined using disc diffusion method according to previously stated protocol [36]. Refreshed culture in TSB (100 µL) was used to inoculate sterile plates in which minimal ISP4 medium was present. Sterile 6 mm filter paper discs impregnated with 5 µL (100 µg/disc) of each test sample were placed on freshly inoculated plates. Surfactin and DMSO loaded discs act as positive and negative controls. The plates were incubated for 3 days so that hyphae formation could take place. Appearance of clear or bald zones around the discs showed the inhibition of hyphae formation. Zones were measured to the nearest mm with a vernier caliper. The sample (daturaolone) which showed ≥12 mm zone of inhibition was tested at lower concentration, i.e., 50, 25 and 12.5 µg/disc, respectively. 

#### 3.5.3. Inhibition of TNF-α-Activated Nuclear Factor-Kappa B (NF-κB)

In the undertaken study, changes in the NF-κB Luc HEK pathway were used to monitor the TNF-α-activated NF-κB inhibitory activity of daturaolone. Stable transfected human embryonic kidney cells 293 (Panomics, Fremont, CA, USA) were employed as reported earlier [39]. Samples presenting greater than 70% inhibitory activity at 20 μg/mL were assessed at three-fold serial dilutions to determine their IC_50_ values. The positive control for this assay was Nα-tosyl-L-phenylalanine chloromethyl ketone (Sigma-Aldrich, aufkirchen, Germany).

#### 3.5.4. Inhibition of NO Production

The NO inhibitory proficiency of daturaolone in murine macrophages was gauged by using previously reported assay procedure [40]. Briefly, for the purpose of primary culturing, primary macrophages obtained from the peritoneal cavity exudates of test animals were washed thrice with FCS-PBS (ice cold). Pre culturing of washed cells was done by making suspension via adding DMEM media for 2 h (37.8 °C, 5% CO_2_) in 24-well microtiter plates.

Afterwards, the non-sticky macrophages were pipetted out and those that sticked to the base of culturing bottle were re culture or reproduced in freshly added DMEM. These adherent cells were then treated with various daturaolone concentrations (20, 10 and 5 µg). Incubation was given for another 120 min. Afterwards, culture was stimulated using LPS (10 mg/mL) followed by additional incubation for one more day. Afterwards, Griess reagent (5% phosphoric acid, 0.1% naphthylethylenediamine dihydrochloride and 1% sulfinamide in ionized water) culture media were intermixed in equal proportion (1:1) were mixed. Finally, absorbance via microplate reader was assessed at 540 nm. Sample which showed than 50% inhibition was tested at lower three-fold dilutions. Table Curve was used to calculate 50% inhibitory concentration, IC_50_.

### 3.6. In Vivo Studies

#### 3.6.1. Dose Selection and Treatment Groups

The doses of daturaolone were selected based on its reported biological activities [5] as well as our results of lymphocyte cytotoxicity and performed acute toxicity studies (data not published yet). Daturaolone 20, 10 and 5 mg/kg were used for subsequent in vivo analysis. A total of six animal groups (I-VI) was made each containing 5 mice (*n* = 5). Groups I-III represented negative control (0.6 mL of normal saline), vehicle control (0.6 mL of 10% DMSO in CMC), and standard control (10 mg/kg of standard drugs) groups respectively. Groups IV-VI represented the test control groups and were administered with 20 (C20), 10 (C10), and 5 (C5) mg/kg of daturaolone respectively. 

#### 3.6.2. Carrageenan-Induced Hind Paw Edema Inflammatory Assay

Daturaolone was checked for its anti-inflammatory property via standard protocol in which carrageenan solution injection is used to induce edema in hind paw (subplantar region) of mice and subsequent measurement of edema thickness by vernier calliper in different test groups reveals anti-inflammatory potential [41]. Briefly, mice were administered 0.6 mL (oral gavage) of the adjusted dose of samples or controls. Time to induce edema via injection (50 μL) of carrageenan suspended in saline was made after one hour of oral gavage of different doses of daturaolone, vehicle, negative and positive controls. Change in paw width was assessed prior to, immediately afterwards and interval of every hour upto 4 h after the carrageenan injection. Resultant paw thickness determination in terms of edema inhibition was assessed using the formula as follows:

Percentage inhibition = [1 − X/Y] × 100 (1)

where “X” is paw edema of treatment and “Y” is paw edema of vehicle control groups. Results are expressed as mean ± SD of % edema inhibition.

#### 3.6.3. Hot Plate Antinociceptive Assay

Thermal pain induction model via standard hot plate was employed to determine antinociceptive response in mice as previously stated and well-established protocol [41]. Saline solution as negative control, 10% DMSO in CMC as vehicle control and tramadol (10 mg/kg) as positive control were utilized. Paw licking and/or jumping response was noted at specified time intervals after placing the mice of respective groups on hotplate. Percentage antinociceptive activity was calculated by the following formula and represented as mean ± SD of % antinociception.

Percentage antinociceptive activity = (Tf − Ti/Ti) × 100 (2)

#### 3.6.4. Tail Suspension Antidepressant Assay

To determine the behavorial antidepressant effect of daturaolone in mice, tail suspension method was adopted as previously stated [42]. Immobility of animal when suspended by tail 7.5 cm above the working table was considered as the sign of depression and was recorded for 6 min. The reduction in the immobility time measured in seconds reflected the antidepressant effect of the treatment. Saline solution as negative control, 10% DMSO in CMC as vehicle control and fluoxetine (10 mg/kg) as positive control were utilized. Results are represented as mean ± SD of immobility time in seconds.

### 3.7. Statistical Analysis

In the undertaken study, the data was statistically tested by using one-way analysis of variance (ANOVA) which was then preceded by Tukey’s multiple comparison test. A *p* value < 0.05 was considered as significant. All the results have been presented as mean ± SD.

## 4. Conclusions

In conclusion, the present study shows that the isolated natural compound daturaolone from *D. innoxia* has drug-like ADME (Pharmacokinetic) properties and almost no toxicity risks predictions. Multiple possible molecular targets were perceived that have important roles in inflammation, cancer, pain and brain disorders. It significantly inhibited the markers of inflammation and cancer, i.e., NF-κB and NO, as reflected by in silico and in vitro models. Furthermore, potent in vivo anti-inflammatory, antinociceptive and antidepressant effects were also observed. On the basis of interrelated in silico, in vitro and in vivo studies, daturaolone is proposed as a drug candidate for tested bioactivities. We suggest detailed pharmacokinetics, toxicity, mechanistic and clinical studies of this natural compound.

## Figures and Tables

**Table 1:** Drug-likeliness, ADME and toxicity prediction of daturaolone.

**Drug Likeliness Profile**											
**^ab^ Lipinski’s Rule**						**Scores**					
MW *	**MLogP**	**N or O**		**NH or OH**		**^b^ Bioavailability**			**^c^ Drug Likeness**		
440.7	5.89	≤10		≤5		0.55			0.33		
**ADME Profile**											
^a^ PPB * (%)	^b^ BBB * **permeability**		^b^ P-gp * substrate		**^b^ Lipophilicity consensus LogP_o/w_**		**^b^ Water Solubility** **LogS** **(ESOL)**		^a^ GI abs * (%)	**^a^ Caco-2 cells permeability** **(nm/s)**	
100	no		no		6.34		Low; −7.29		High: 96.6	34.6	
**Toxicity Profile**											
**^a^ Ames Test**					^a^ hERG * inhibition risk			**^a^ Carcinogenicity**			
Pred *	TA100 *		TA1535 *					Mouse		Rat	
	**+S9**	**−S9**	**+S9**	**−S9**							
Non-mutagen	-ive	-ive	-ive	-ive	Low			No		No	

Data was predicted from; ^a^ = PreADMET, ^b^ = SwissADME and ^c^ = MOLSOFT. * MW = molecular weight; * P-gp = P-glycoprotein; * GI abs = gastrointestinal absorption; * Pred = prediction; -ive = no toxicity prediction for Ames test; * hERG = human ether a go go related gene. * TA 100 and * TA 1535 are test strains used for Ames test. +S9 and −S9 shows with and/or without metabolism of tested compound.

**Table 2:** Predicted biological effects, associated molecular descriptors and cytotoxicity prediction against cancer cell lines of daturaolone.

**^a^ Anti-Inflammatory**						
^a^ Nuclear factor kappa B	^b^ NO synthase	^b^ Arachidonate-5 lipoxygenase	^b^ Cyclooxygenase 2		^b^ Phospholipase A2	
**^a^ Antinociceptive**						
^b^ Prostaglandin E synthase	^b^ Peroxisome proliferator-activated receptor-alpha	^b^ 5-HT receptor 1B	^c^ Estrogen receptor antagonist		^b^ Cannabinoid receptor 1	
**^a^ Antidepressant**						
^b^ 11-betahydroxysteroid dehydrogenase 2	^b^ Norepinephrine transporter	^c^ Dopamine 1A receptor antagonist	^c^ GABA transporter 1 antagonist		^b^ Serotonin transporter	
**^d^ Cancer cell lines cytotoxicity prediction (Pa > Pi)**						
**Leukemia**	**Pancreatic carcinoma**	**Thyroid carcinoma**	**Gastric carcinoma**	**Hepatoblastoma**		**Melanoma**
HL-60	PANC-1	8505-C	MKN-7	HepG2		SK-MEL-1
0.53 > 0.02	0.48 > 0.00	0.47 > 0.00	0.43 > 0.00	0.42 > 0.03		0.48 > 0.03

Prediction done via PASS online (^a^), SWISS target prediction (^b^), GUSAR antitargets (^c^) and PASS CLC Pred (^d^). Pa and Pi are the probability of substance being active and non-active in respective mode.

**Table 3:** In vitro cytotoxicity assessment of daturaolone in cancer cells after 72 h and in normal isolated lymphocytes after 24 h.

In Vitro Cytotoxicity Assessment in Cancer and Normal Cells							
Cell lines	C20		Vincristine		Cabazitaxel		1% DMSO
	% Inhibition	IC_50_ (µg/mL)	% Inhibition	IC_50_ (µg/mL)	% Inhibition	IC_50_ (µg/mL)	% Inhibition
Huh7.0	38.33 ± 1.58	>20	86.75 ± 0.56	6.75 ± 0.79 ^a^			--
Huh7.5	53.60 ± 3.24	17.32 ^b^ ± 1.43	84.57 ± 0.49	5.62 ± 0.72 ^a^			--
DU145	54.70 ± 2.21	18.64 ^b^ ± 2.15			94.75 ± 0.15	4.75 ± 1.12 ^a^	--
PC3	41.53 ± 2.36	>20			92.76 ± 1.76	6.62 ± 2.11 ^a^	--
MCF7	46.86 ± 4.66	>20	78.55 ± 2.36	4.33 ± 1.87 ^a^			--
Lymphocytes	29.13 ± 2.57	>20	67.73 ± 1.70	6.86 ± 0.49 ^a^			--

C20 (Daturaolone), cabazitaxel, vincristine and 1%DMSO are sample, positive control reference standards and negative controls, respectively. Daturaolone and reference/positive controls were tested at 20 µg/mL. After triplicate (*n* = 3) performance of experiment, values were calculated as mean ± standard deviation. No cytotoxicity or no activity was seen (--). Statistically significance is represented as alphabets a–c, which means difference is highly significant, slightly significant and significant at *p* < 0.05, respectively. 1% DMSO did not show any cytotoxicity in cells.

**Table 4:** Protein kinase inhibition, TNF-α activated NF-κB inhibition and NO production inhibition potential of daturaolone.

Sr. No.	Sample	Protein Kinase Inhibition			TNF-α-Activated NF-κB Inhibition		Inhibition of NO Production	
		Clear Zone (mm)	Bald Zone (mm)	MIC (µg/disc)	% Inhibition	IC_50_ (µg/mL)	% Inhibition	IC_50_ (µg/mL)
1	**C20 (µg/disc)**	10.22 ± 1.74	12.37 ± 2.41 ^b^	20				
2	**C20 (µg/mL)**				92.17 ± 5.1 ^a^	1.2 ± 0.8 ^a^	84 ± 2.87 ^a^	4.51 ± 0.92 ^a^
3	**Surfactin**	0	28.23 ± 2.63 ^a^	-				
4	**TPCK**				87.23 ± 4.38 ^a^	10.7 ± 1.6 ^a^		
5	**Curcumin**						91 ± 3.31 ^a^	2.94 ± 0.74 ^a^
6	**≥1% DMSO**	-	-	-	-	-	-	-

Values are presented as mean ± standard deviation (*n* = 3). (-) No inhibition. Surfactin (20 µg/disc), curcumin (20 µg/mL), and Nα-tosyl-L-phenylalanine chloromethyl ketone (TPCK; 20 µg/mL) were used as positive controls of protein kinase inhibition, NO inhibition and TNF-α activated NF-κB inhibition assays, respectively. None of the test samples exhibited cytotoxicity at the IC_50_. ≥1% DMSO was used as negative control and did not exhibit any activity. Exclusion of false positive results mandated the performance of parallel cytotoxicity assay. None of the test samples demonstrated cytotoxicity at the IC_50_. a,b shows if difference is significant or not at *p* = 0.05.

## Data Availability

Data is contained within the article and Supplementary Materials.

## Supplementary Materials

The following are available online at https://www.mdpi.com/article/10.3390/ph14121248/s1. Figure S1a: Isolation scheme of daturaolone. Figure S1b: GC-MS spectra of daturaolone. Figure S1c: 13C-NMR. Figure S1d: 1H-NMR. Figure S1e: HSQC NMR. Figure S1f: HMBC NMR. Figure S1g: COSY NMR. Figure S1h: Elucidated structure of daturaolone. Figure S1i: HPLC DAD Chromatogram of daturaolone. Figure S2: Drug likeliness prediction by molsoft with score of 0.33. Figure S3: Protein kinase (PK) inhibitory potential of daturaolone (20 ug/disc). Table S1: ADME data table of Daturaolone from SWISS ADME.
