# Supplementary material for: Anti-Inflammatory Potential of Daturaolone from Datura innoxia Mill.: In Silico, In Vitro and In Vivo Studies
Source: Pharmaceuticals (Basel). 2021 Nov 30;14(12):1248. doi: 10.3390/ph14121248 (PMC8708807; doi:10.3390/ph14121248)
Supplement: Supplementary file 1 [file pharmaceuticals-14-01248-s001.zip › pharmaceuticals-1434176-supplementary.pdf]

pharmaceuticals

Supplementary materials

Anti-Inflammatory Potential of Daturaolone from *Datura innoxia* Mill.: In Silico, In Vitro and In Vivo Studies

Muhammad Waleed Baig [1], Humaira Fatima [1], Nosheen Akhtar [2], Hidayat Hussain [3], Mohammad K. Okla [4], Abdulrahman Al-Hashimi[4] Wahidah H. Al-Qahtani [5], Hamada AbdElgawad [6] and Ihsan-ul Haq [1,*]

[1] Department of Pharmacy, Quaid-i-Azam University, Islamabad 45320, Pakistan; mwbg7@yahoo.com (M.W.B.); hfchughtai@qau.edu.pk (H.F.)
[2] Department of Biological Sciences, National University of Medical Sciences, Rawalpindi 46000, Pakistan; nosheenakhtar@numspak.edu.pk
[3] Leibniz Institute of Plant Biochemistry, Department of Bioorganic Chemistry, Weinberg 3, D-06120 Halle (Salle), Germany; hidayat.hussain@ipb-halle.de
[4] Botany and Microbiology Department, College of Science, King Saud University, P.O. Box 2455, Riyadh 11451, Saudi Arabia; malokla@ksu.edu.sa (M.K.O.); alhashimi@ksu.edu.sa (A.A.-H.),
[5] Department of Food Sciences & Nutrition, College of Food & Agriculture Sciences, King Saud University, Riyadh 11451, Saudi Arabia; wahida@ksu.edu.sa (W.H.A.-Q.)
[6] Integrated Molecular Plant Physiology Research (IMPRES), Department of Biology, University of Antwerp 2020, Groenenborgerlaan 171, Antwerp, Belgium; hamada.abdelgawad@uantwerpen.be (H.A.)
* Correspondence: ihsn99@yahoo.com; Tel.: +92-321-5123877

Citation: Baig, M.W.; Fatima, H.; Akhtar, N.; Hussain, H.; Okla, M.K.; Al-Aashimi, A.; Al-Qahtani, W.H.; AbdElgawad, H.; Haq IU. Anti-Inflammatory Potential of Daturaolone from *Datura innoxia* Mill.: In Silico, In Vitro and In Vivo Studies. *Pharmaceuticals* **2021**, *14*, x. https://doi.org/10.3390/xxxxx

Academic Editors: Dejan Stojković and Marina Sokovic

Received: 19 October 2021
Accepted: 23 November 2021
Published: 30 November 2021

Publisher's Note: MDPI stays neutral with regard to jurisdictional claims in published maps and institutional affiliations.

Copyright: © 2021 by the authors. Submitted for possible open access publication under the terms and conditions of the Creative Commons Attribution (CC BY) license (http://creativecommons.org/licenses/by/4.0/).

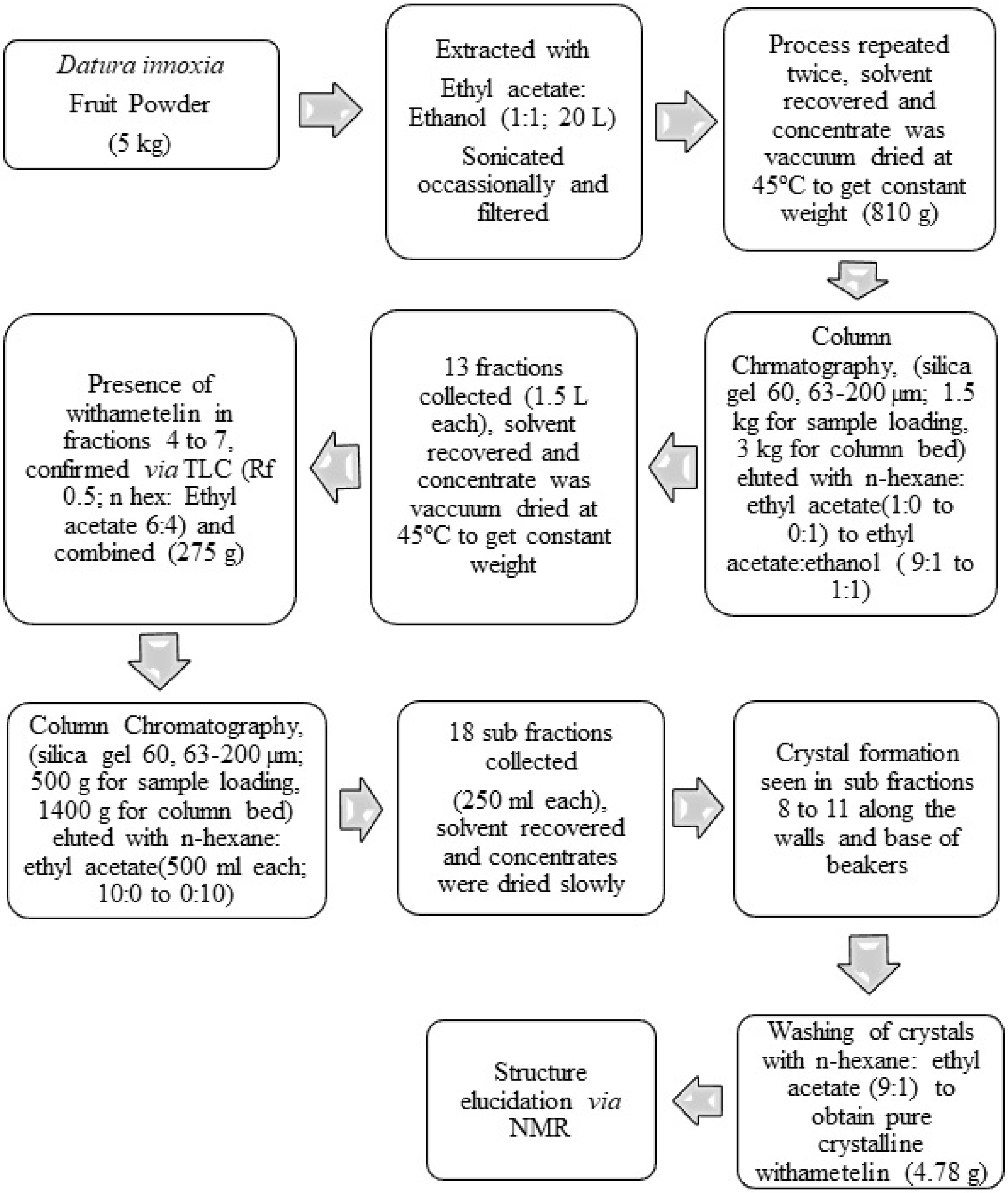

Figure S1.a. Isolation scheme of daturaolone.

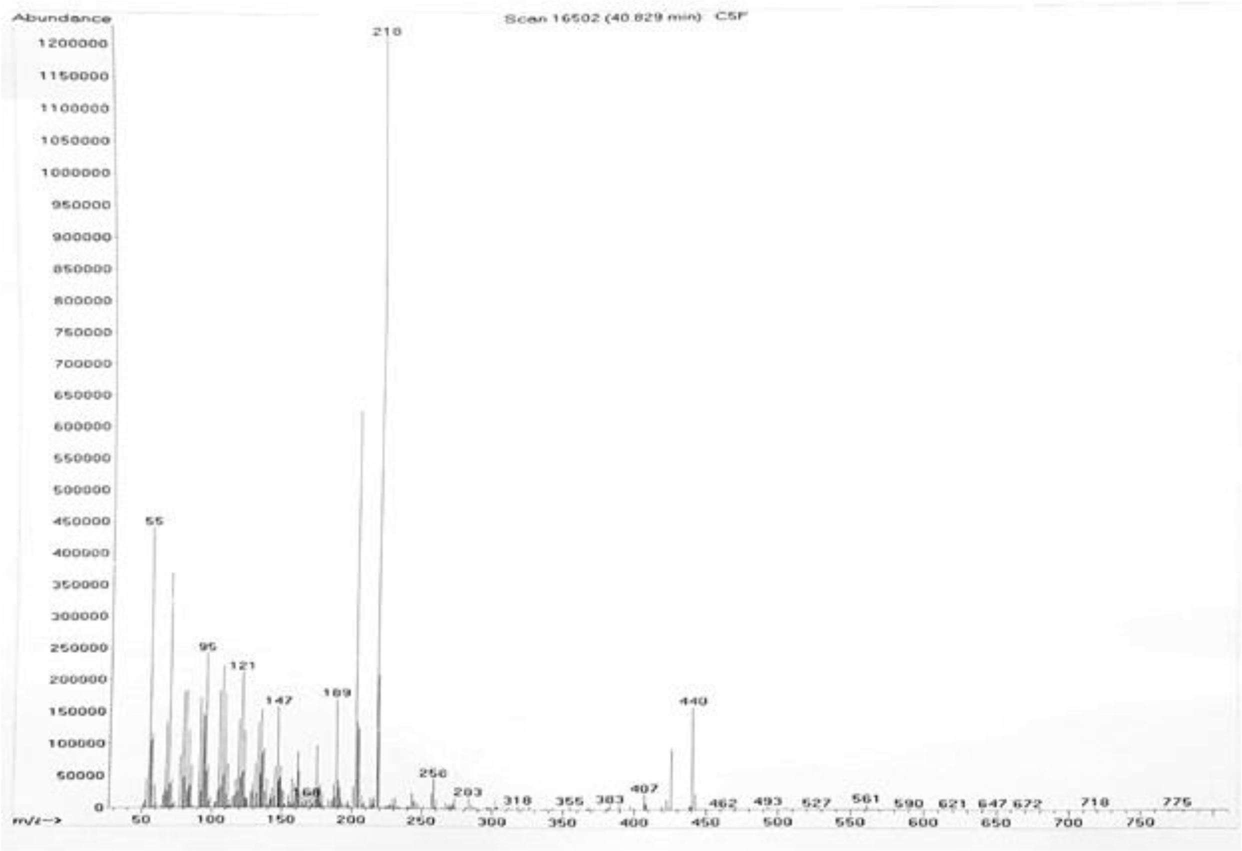

Figure S1b. GC-MS spectra of daturaolone

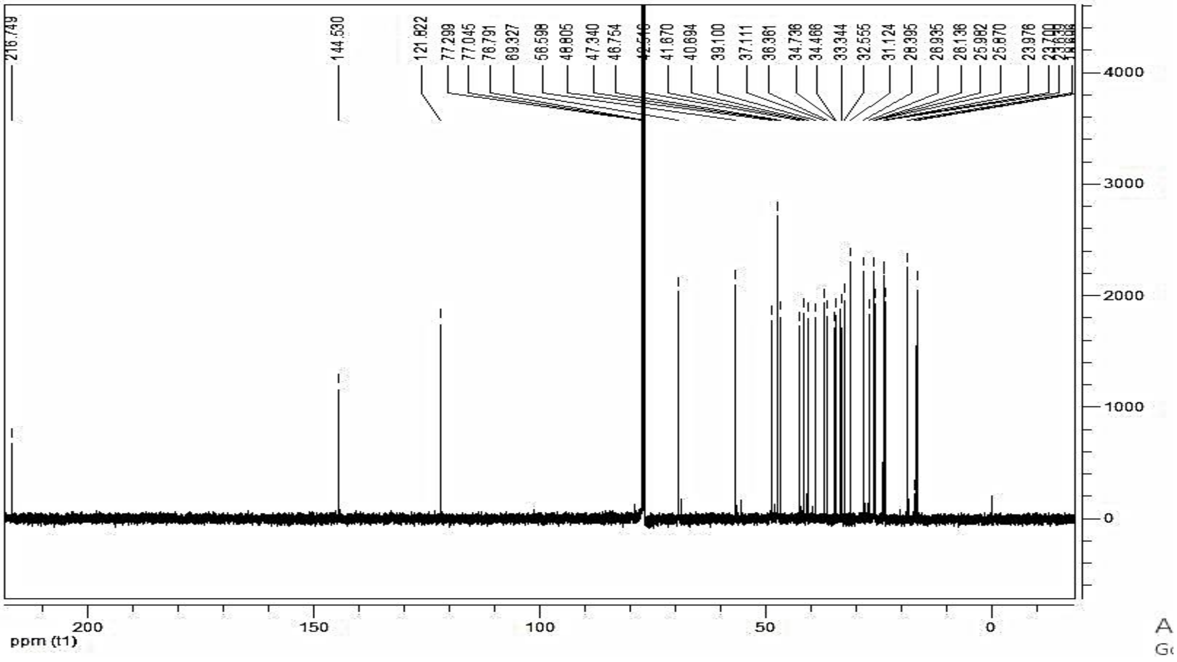

Figure S1c. ^{13}C-NMR

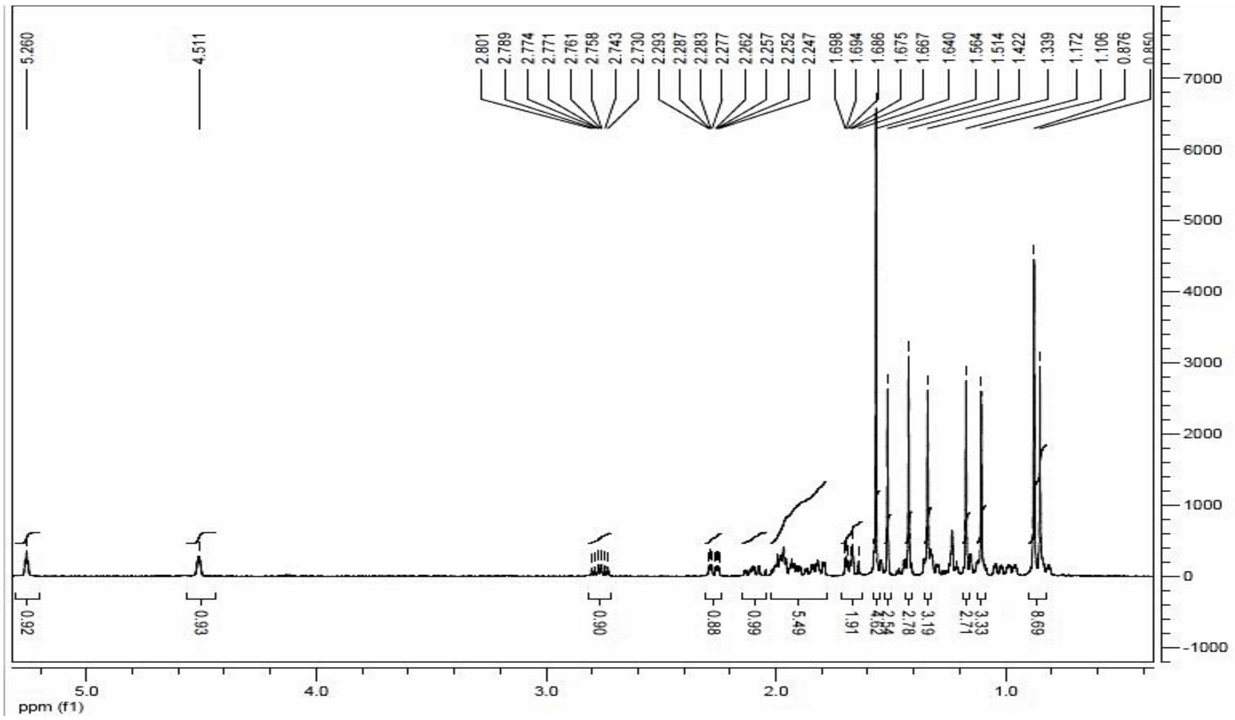

Figure S1d. 1H-NMR

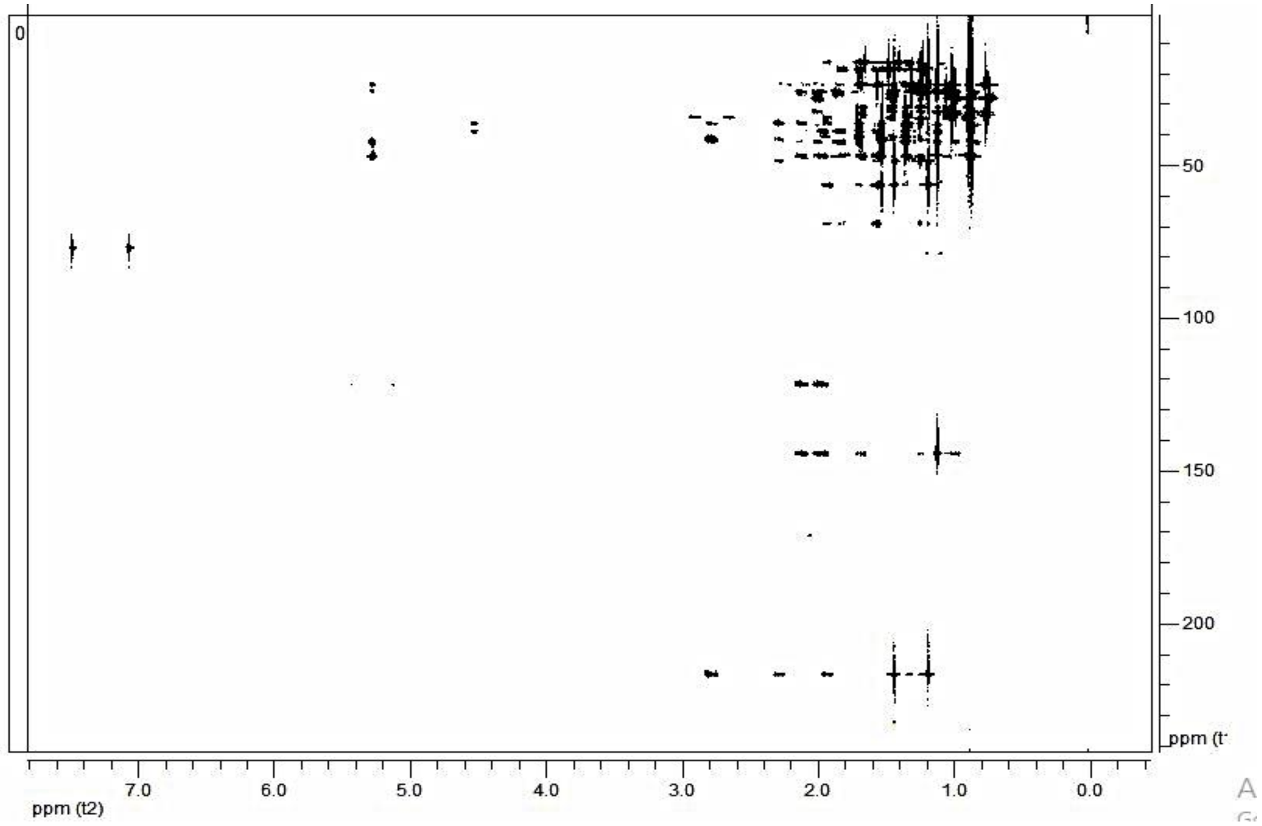

Figure S1e. HSQC NMR

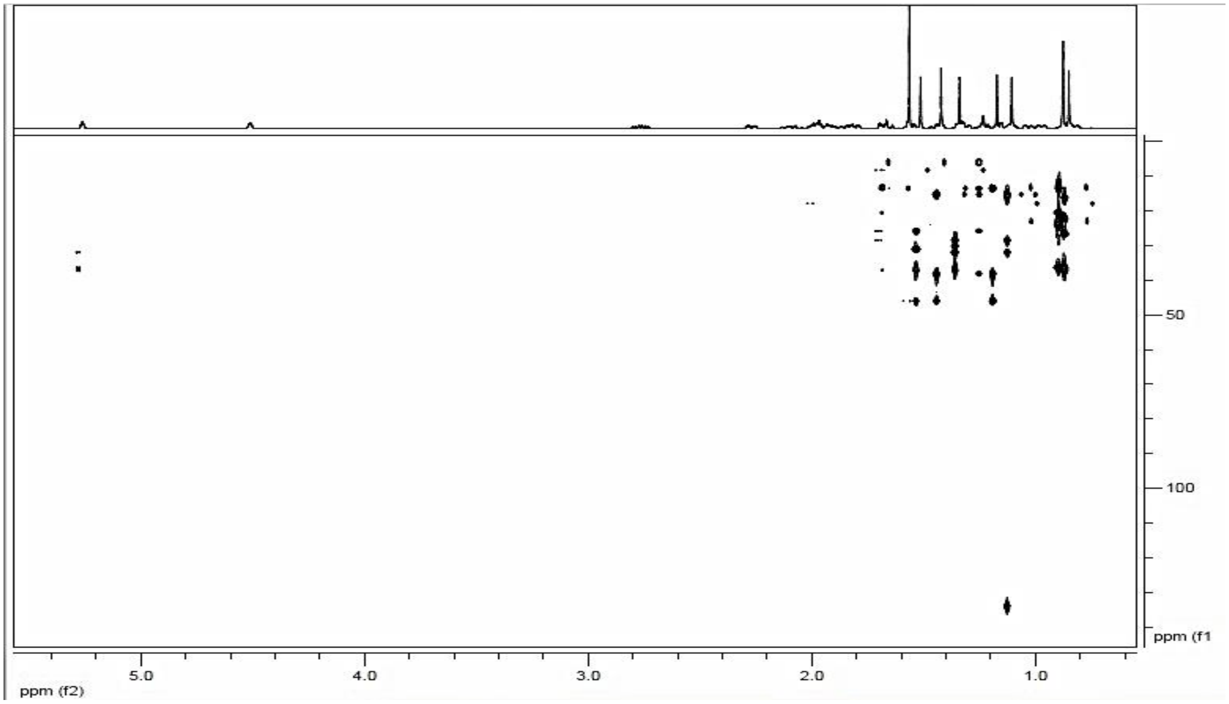

Figure S1f. HMBC NMR

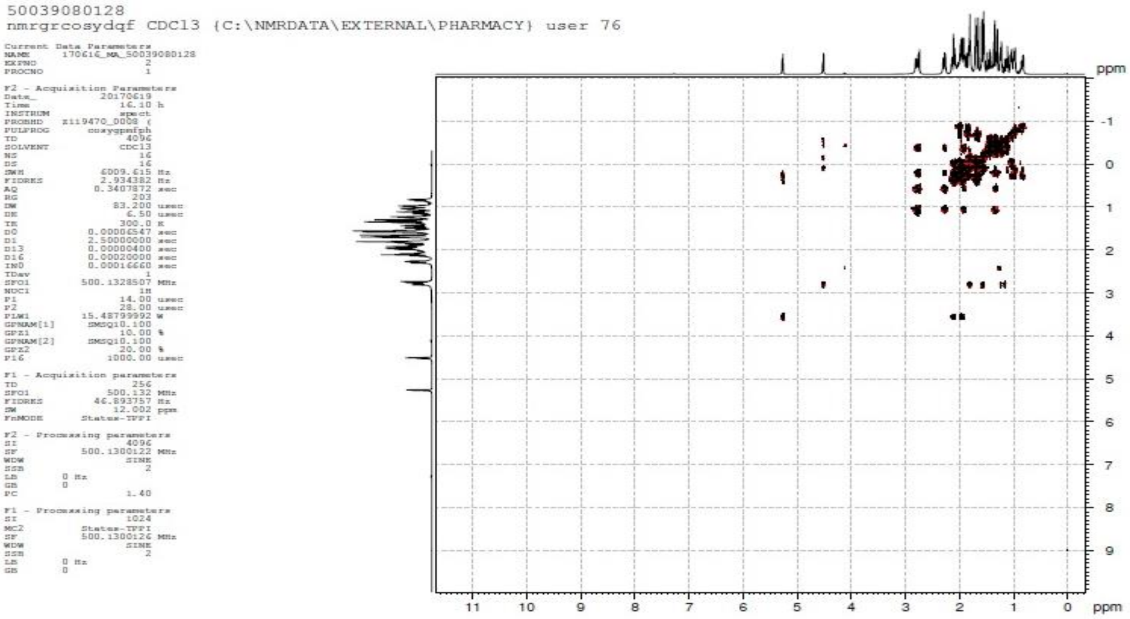

Figure S1g. COSY NMR

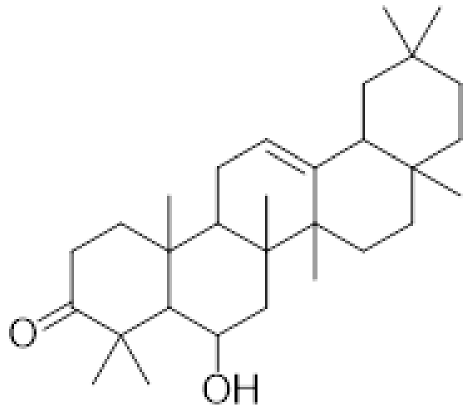

Figure S1h. Elucidated structure of daturaolone

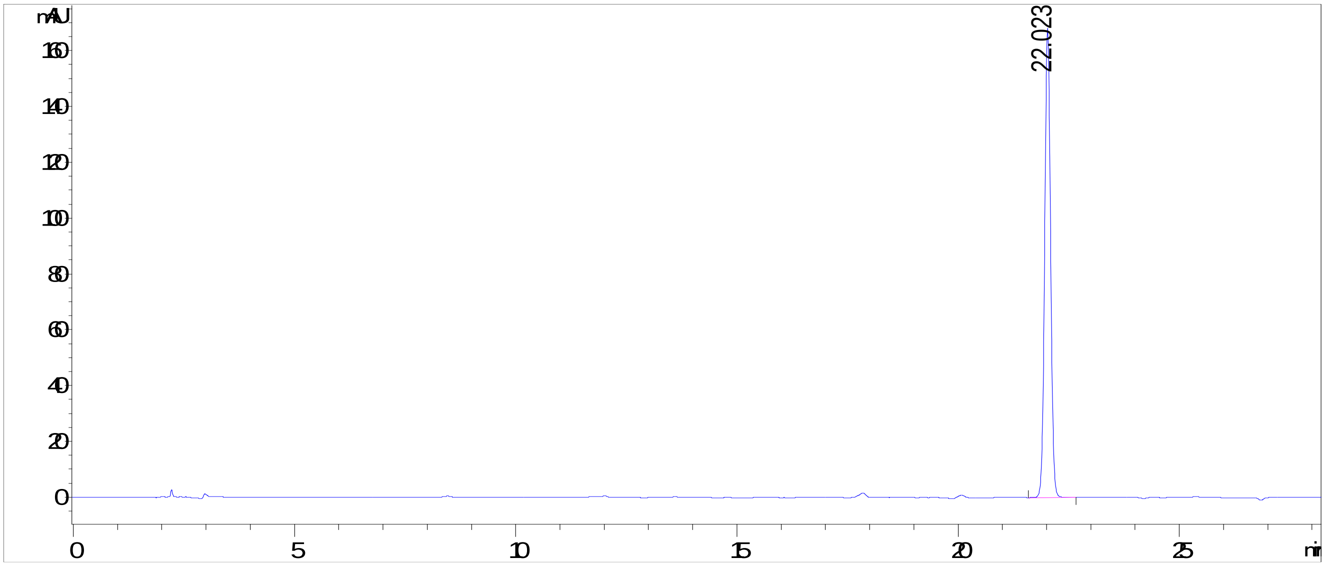

Figure S1i. HPLC DAD Chromatogram of daturaolone.

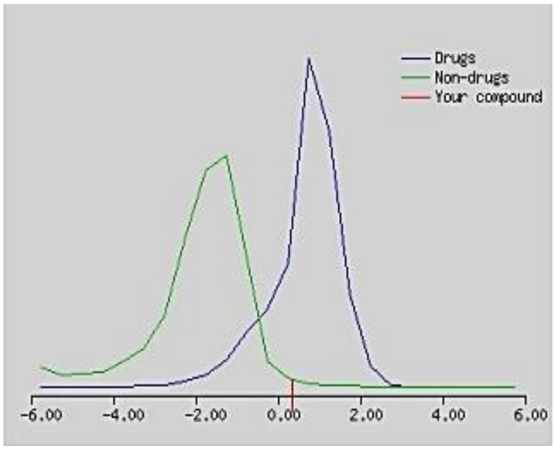

Figure S2. Drug likeliness prediction by molsoft with score of 0.33

Table S1. ADME data table of Daturaolone from SWISS ADME

Molecule	Daturaolone
Canonical SMILES	OC1CC2(C)C(C3(C1C(C)(C)C(=O)CC3)C)CC=C1C2(C)CCC2(C1CC(CC2)(C)C)C
Formula	$C_{30}H_{48}O_2$
MW	440.7
#Heavy atoms	32
#Aromatic heavy atoms	0
Fraction Csp3	0.9
#Rotatable bonds	0
#H-bond acceptors	2
#H-bond donors	1
MR	135.08
TPSA	37.3
iLOGP	4.37
XLOGP3	7.49
WLOGP	7.35
MLOGP	5.89
Silicos-IT Log P	6.61
Consensus Log P	6.34
ESOL Log S	-7.29
ESOL Solubility (mg/ml)	2.25E-05
ESOL Solubility (mol/l)	5.12E-08
ESOL Class	Poorly soluble
Ali Log S	-8.11
Ali Solubility (mg/ml)	3.45E-06

Ali Solubility (mol/l)	7.82E-09
Ali Class	Poorly soluble
Silicos-IT LogSw	-7.04
Silicos-IT Solubility (mg/ml)	4.02E-05
Silicos-IT Solubility (mol/l)	9.12E-08
Silicos-IT class	Poorly soluble
BBB permeant	No
Pgp substrate	No
Lipinski #violations	1
Bioavailability Score	0.55
PAINS #alerts	0
Brenk #alerts	1
Leadlikeness #violations	2
Synthetic Accessibility	5.95

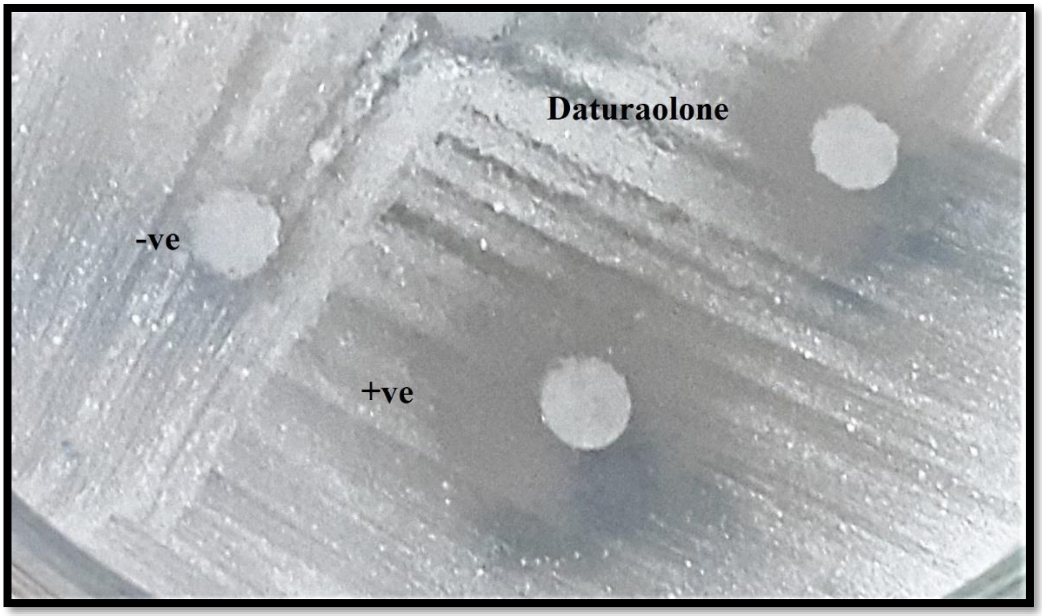

Figure S3. Protein kinase (PK) inhibitory potential of daturaolone (20 ug/disc). Surfactin infused disc was used as positive control and DMSO infused disc was used as negative control.
